# Reflection on traditional sporting games: the impact of bodily involvement on empathic dimensions

**DOI:** 10.3389/fpsyg.2023.1123519

**Published:** 2023-05-30

**Authors:** Eric Dugas, Boubaker Ben Ali

**Affiliations:** ^1^LACES UR 7437, University of Bordeaux, Bordeaux, France; ^2^ECOTIDI UR 16ES10, Virtual University of Tunis, Tunis, Tunisia

**Keywords:** empathic dimensions, internal logic motricity, decision-making, interaction, traditional sporting games

## Abstract

The aim of this study was to investigate bodily engagement and involvement in traditional sporting games (TSGs), with a focus on the development of empathy. Even though the current research on empathy has been focused on its emotional component, the name “empathy” alludes to a considerably more profound dimension than emotional engagement. Empathy refers to the ability to perceive another person’s private life through the exchange of contextual factors provided through interactive sports activities. In this study, based on real-world experiences, it has been demonstrated that traditional sporting games stimulate, preserve, or reveal empathic capacities in several ways. Games can show and sustain the full potential of empathic dispositions if they are present at a young age. Moreover, by examining empathy through the prism of a TSG, we recognized them as a source of relational empathy and feelings developed to various degrees by direct involvement. As a result, we may define empathy as an integrated pedagogy that can be more successfully conducted through TSGs which are multifaceted because of their internal and external logic systems. Essentially, the hypotheses discussed in this study allow us to postulate that the physical gaming involvement of players, such as role changes, influences the individual’s empathic dimensions. Furthermore, the characteristics of traditional sporting game interaction networks may serve as a source of encouragement or inspiration for a wide range of games (theatrical, social, etc.).

## 1. Introduction

Throughout history, the definition of “gaming” has shifted from potentially educational or social activities to undesirable or futile activities reflecting deviant behaviors (Brougère, 1995, 2012). Previous centuries of research have shown that games, even so-called sporting games, were the target of criticism and disrespect (Parlebas, 1975, 1995; During, 1981; Harouel, 2011). As a result, many academic researchers could not see games as a worthy topic of study, which is why there was considerable interest in other topics such as emotion or ideology. However, as Parlebas and Boutin (2022) highlighted in their study, some well-known scientists preferred studying games despite their appearances, misunderstandings, and the controversies surrounding them. Several interesting studies have been published, including Jérôme Cardan’s “Hazard Games” (Morley, 1854), Von Neumann’s “Social Games” (von Neumann and Morgenstern, 2004), Bernoulli’s (1713) “Palm Game and Mathematical Expectation,” Borel’s “Poker Game” (Ferguson, 2003), the famous works of Caillois (1955) and Winnicott (1971) about games and reality. Within the cited categories of investigations, this study focuses on traditional sporting games (TSGs).

Since TSGs are non-institutional sporting events, undoubtedly, they allow for outstanding interactional frameworks (Parlebas, 1981, 2020; Dugas, 2004). These codified ludomotor activities may reveal their merits through the original physical interactions in which we live with ourselves and others (partners or opponents), which may stimulate and strengthen all players’ empathetic behavior. Indeed, physical play could promote the development of empathy by encouraging “daring” and “feeling the other.” Empathy is a necessary component of human relationships and social existence. It is multidimensional, involving “affective empathy, cognitive empathy, concern for others, emotional regulation, and self-awareness” (Decety, 2020). As a result, it is critical to distinguish all empathy factors from other human qualities. Thus, one point in common between the concepts of “empathy” and “game” is their amorphous and non-conformist nature. This peculiarity has resulted in a demultiplication of recent academic works incorporating new points of view (Attigui and Cukier, 2019). Tisseron and Bass (2011) defines empathy as a necessary component and as an unavoidable cement of human relationships.

Professionals in the workforce, care, management, and education stick to new ideas like “care,” “wellbeing, and “professional empathy” in the face of “ill-being” (Dugas et al., 2020); therefore, a large amount of research has been focused on the “development of psychosocial skills” (OMS, 1997), which is essential to any social life. Furthermore, the recent COVID-19 pandemic has aggravated this phenomenon at the heart of society, businesses, and schools (Dugas et al., 2020) by slowing the emergence of a new awareness of a world in crisis (Rifkin, 2011). As a result, a deep understanding of the required coupling between “person and environment” is necessary, along with a meticulous examination of the structures through which the players move.

The spaces where we live, learn, and work are more than just decorations (Moser, 2009); they shape our attitudes and actions (Fischer, 2011). Thus, the relationship between “player(s) and game structures” is therefore interesting to investigate, since it may lead us to question and analyze how TSGs (Lavega et al., 2014; Lavega-Burgués et al., 2020; Parlebas, 2020) may act as a lever for relational empathy. Furthermore, it also led us to question how it could function as an operational and dynamic revealer of empathy built through playful interactions. In this context, games may be an effective means of cultivating empathy in individuals and groups (Decety and Cowell, 2014).

This study examines the relationship between “player(s)/game structures” and empathy through games with interdependent ludomotor interactions (Dugas, 2011a). It offers an opportunity to enhance our knowledge concerning the concepts of “empathy” and “traditional sporting games” through the prism of motor praxeology and a systemic approach (Parlebas, 1981, 1999). It also analyzes several field studies associated with TSG to feed our reflection that these sporting games are likely to stimulate, maintain, or reveal in various ways the potentialities and empathic capacities of those players (Lavega et al., 2014; Lavega-Burgués et al., 2020; Parlebas, 2020). Finally, the impact of bodily involvement can inspire other forms of games to stimulate empathetic awakening.

Throughout this study, we attempted to investigate through examples how a TSG, as a codified ludomotor activity, might reveal all its attraction through the original physical interactions in which we, that is, ourselves and others (partners or opponents), live. The main aim of this study was to unravel how gaming stimulates and strengthens the empathetic conduct of players through physical involvement. We also analyzed the question “how could the internal logic of a game affect all aspects of the players and enhance their emotional contact and resonance (Zanna, 2015b; Zanna and Jarry, 2019) by mobilizing their empathetic conduct and availability?”

Indeed, to encourage the development of empathy by “daring” and “feeling the other” through physical play, we also put forward that a complete understanding of the prerequisite coupling between “person and environment” is required. Therefore, a meticulous examination of the structures through which the players move seems to be crucial (Dugas, 2011b; Dugas and Loyer, 2018; Parlebas, 2020), because while there is undeniably a “Me” in “Game,” there is also a “Game” in “Me” (Parlebas, 1975).

## 2. Systemic approach: differences between sporting games and other games

Let’s start with a study of ludomotor activities that need medium to fine motor skills (such as TSG, leisure games, and competitive games) because they are commonly practiced (Parlebas, 1981). These activities are opposed to motor skills at work (ergomotricity) and games devoid of motor relevance such as cognitive games (or board games). In this regard, Pierre Parlebas considers that, in games of chess, bridge, or scrabble, “[…] relevance is not driving but combinatorial and/or symbolic.” These activities are therefore not sporting games according to the point of view adopted.

In addition, various sorts of games (Berry, 2012) have appeared in modern societies, such as computer games, serious games, and other escape games that need motricity to some level. Thus, the controller held in the hand can replace the player in tennis or a team in a football match. However, it is inconceivable to confuse a tennis controller-handling player with a racket-handling athlete (Bordes et al., 2007).

Motor abilities, in summary, might be actual, mimicked, or even virtual, since they are becoming increasingly associated with virtual reality (VR) or augmented reality as technology advances (Tassinari et al., 2021). In addition, it is possible to project oneself into a world where players remain on the edge of ludomotor reality, for example, using artificial sensory systems (Ben Ali et al., 2018, 2020a) of the main human senses (such as sight, hearing, and touch). However, this ludomotricity does not combine (yet) the physical and biological constitutions of the sporting factors and has no real motricity (Delaunay, 1981).

The space of ludomotor practices extends from unorganized and free activities to formal and institutionalized activities, called “sports” by Parlebas (2010). Between the two poles of this ludomotor chain, we found games that are not under the supervision of a sports federation. However, these physical games outside institutions are codified and endowed with rules shaping the practitioner’s confrontation with the human and/or physical environment. Among these rich and varied practices, we found TSGs (Dugas and During, 2006).

Faced with this proliferation of ludomotor practices, this article focuses, as mentioned above, on sporting games, defined as a “codified motor situation of confrontation, called games or sports by social authorities”.

**Figure 1:**
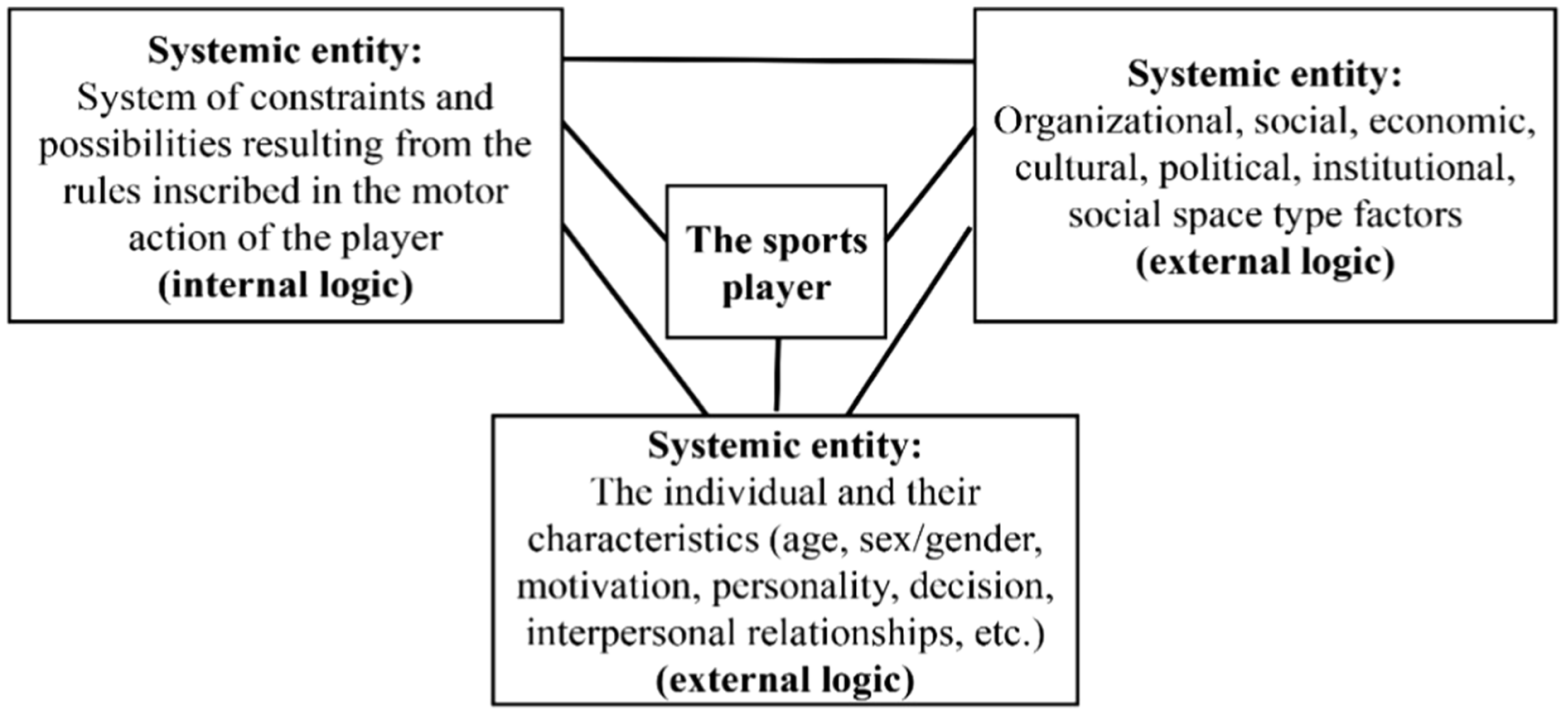
Systemic approach to internal and external logics to make player behavior intelligible; adaptation of the diagram from *L’homme Systémique* (Dugas, 2011b).

A sporting game is defined by its system of rules that determines its internal logic (Parlebas, 1999). Sporting games are thus accomplished based on a ludic contract within which motor practice is subject to a system of rules that makes it meaningful. From then on, the sporting game is at the crossroads of collective rules and individual choices, and once the latter are subject to the former, the game acquires the role of participating in culture and education. Moreover, this ludic context allows many types of ludomotor interactions that do not take place in a social vacuum. The behaviors of the players are thus placed at the heart of the system in an inseparable relationship with the context of action, having original problems to solve. It is, therefore, recognized as a “praxeological” system (Parlebas, 1986) before understanding the dynamics of empathy that we are going to explore in this study. In our view, this systemic approach is composed of three interacting “systemic entities’, as shown in Figure 1.

In 1976, Parlebas (1976, 2020) stated that “behind the superficial disorder that is all the rage in traditional games, there is an in-depth order in there too.” This remark recalled two inextricably linked, interconnected, and fundamental logics of the environments in which the actors’ conduct occures. Parlebas defines them as universals,” since these rules or subordinate objective systems serve as a basis for the “praxic exchanges” observed in all games and sports.

- An “internal logic” to the situation, presenting a system of constraints and possibilities resulting from the rules that are part of the playful action of the players and that take into account the relationship of the intervener to the physical environment and to the other characteristics such as objects, time, space, and the scoring system (if present). This first logic is defined by the “system of relevant features of a motor situation and the consequences it entails in the performance of the corresponding motor action” (Parlebas, 1999).
- An “external logic” divided into two systemic variables: the characteristics of the actors (e.g., age, sex/gender, otherness, personality, motivation, mobilization, and interindividual relations) and the organizational characteristics (e.g., social factor, socioeconomic, cultural, political, institutional, or social spaces). Moreover, many other factors can also intervene within the external logic (e.g., institutions, cultural belonging, group dynamics, relational aspects, the motor intelligence of the individual, and their empathy capacities).

Each sporting game stages its own universal steps, leading to extremely varied specific behaviors, extraordinarily rich in relational consequences. The “universals” of team sports show a great relational clarity that makes them a reference of undeniable interest. For example, team sports only accept symmetrical (football) or asymmetrical (baseball) duels between two teams with stable relations between partners and adversaries, while the salient rules of TSG allows us to compare the variety of motor interactions allowed by the networks of exchanges. This category of games will be of particular interest to us as a lever for empathy.

## 3. Traditional sporting games as a backdrop for an empathic plea

The desire to oppose, win, or dominate informs us about the cultural background of civilizations. Indeed, not all games are strictly competitive in every society (Lévi-Strauss, 1962). Many sporting games are unconventional in their relationships with machines, equipment, and space, especially in their relationship with others (e.g., relational structures such as “each for themselves,” “one team against others,” “one against all,” “paradoxical games,” “determined games,” “rite games,” etc.).

Thus, unlike team sports (e.g., football, handball, volleyball, rugby, and basketball), many traditional collective sporting games and some situations of opposition do not systematically seek victory and domination over others. Thus, relationships among players can be stable or unstable (Parlebas, 2020). Players can change teams or alliances if there are no predefined teams.

Indeed, the relational network in these games is permutable and dictated by the rules of the game. Moreover, with each winning interaction of one of the players, the players change teams or roles. The presence of this interactional configuration in the games is presented in Section 3 of this article.

In strictly competitive games (institutional duels), relational empathy does not involve decoding the other or others to be effective (as in animal ethology). Thus, in dueling sports, game rules regulate the flow of empathy with the aim of utility or gain. It is preferable neither to have positive feelings for the opponent nor to be overwhelmed by emotions. Ruse, deceit, bodily manipulation (feint, lure), and verbal manipulation are therefore crucial in these encounters. We regularly hear athletes say that, if friendship persists, it is left in the locker room. Empathy and understanding of the other are self-serving and non-communicative.

In traditional games, relational networks are sometimes ambivalent, depending on the choice of players. The player can choose to team up or oppose. This is the sphere of “paradoxical games”—a special concept that belongs to a category of games that increases the originality and complexity of the social bond. These are so-called paradoxical games that immerse players in an ambivalent network such that each player is at the same time adversary and partner of any other participant (Parlebas, 2010).

### 3.1. Empathy at the heart of sporting games

Empathy may be assessed and improved through physical games. Indeed, the modern monistic conception of the person encompasses all human relationships. What could be simpler than using ludomotor games to achieve that goal? “To be able to engage into emotional empathy, you need presence, you need body in a space–time that supports direct face-to-face,” as Zanna and Jarry (2019) stated. Furthermore, according to Goffman, “a well-aware person who wishes to avoid emotional overflowing needs to engage in cognitive reflexivity” (Warnier, 1999).

Empathy is a concept that has come to the fore in the scientific literature in recent years. However, we should not forget that it is protean, subject to controversy, praised or, on the contrary, subject to suspicion. The effect of fashion has contributed to a magnifying glass effect on the concept of empathy by enlarging the positive or negative traits and the advantages and the disadvantages. However, the specialness of empathy is to be multidimensional.

The concept of empathy has its roots in Robert Vischer’s works from the nineteenth century, which emphasized a feeling that arises within the context of esthetic experience (Einfühlung). According to Pacherie (2004), it is the ability to comprehend what another person is thinking and feeling while keeping in mind that the other person is not the same as the individual. In fact, it makes sense to employ the “as if” idea from the Rogerian method to better describe that sentiment/circumstance. In addition, research supports a variety of forms of empathy that are linked to both main components: emotional and cognitive affection (Decety and Jackson, 2004; Batson, 2009).

### 3.2. Empathy as a multidimensional concept

The primary characteristics of empathy may be agreed upon from the expression: “Empathy has two faces, like the god Janus of antiquity” (Tisseron 2011). On the one hand, it allows us to have a mental representation of the mental and affective functioning of our interlocutor; on the other hand, it brings us into resonance with sensory and emotional states.” Empathy thus has two aspects—cognitive and affective emotion. In other words, it has two interacting elements (Decety, 2010): (1) a more automatic and hardly intentional motor resonance and (2) a more controlled and intentional subjective perspective of others. Zanna and Jarry (2019) summarized these two dimensions when a situation finds at least two people in interaction: cognitive empathy allows us to understand the other and to represent him or her to oneself, while emotional empathy is a face-to-face relationship where we resonate with the other (through the body and language) while maintaining the “right” distance so that he or she need not be emotionally confused. This subtly differentiates empathy from sympathy and compassion (feeling the pain of others for the sake of the other). In short, for Zanna (2015a), as for other specialists, empathy offers the discovery of “another possible self.”

Cognitive empathy cut off from its emotional dimension (e.g., having no need to experience one’s emotions) can be a dangerous weapon that is used to manipulate others for one’s own ego (maximizing one’s gains, pleasure, or enchantment at the expense of the other). The risk is also to be overwhelmed by one’s emotions, which can lead to passivity, refusal, or denial (of the other), depending on the situation experienced. Finally, bias can occur through empathy and interfere with moral behavior, especially by favoring one person or group (Decety and Cowell, 2014).

Thus, in the literature, it has been argued that while empathy can generate risks, the absence or lack of empathy is conducive to deviant, inappropriate, or violent behavior. It can even lead an individual to mistreat, harass, or violate others (Dugas, 2020). However, it can also be beneficial in certain circumstances: “Imagine a surgeon operating on a loved one! Better to cut yourself off from such emotional empathy in such a situation, although only for a while.”

Finally, we should keep in mind that empathy is mature “if there is an intentional solicitation of her two components, by adopting the affective perspective of others, for instance” (Decety, 2020). Advances in knowledge, especially knowledge linked to social neuroscience, reveal that empathy is a disposition specific to humans from an early age. Certain brain regions are responsible for morality and our moral sense. Among other human qualities, this empathic predisposition plays an important role in the subsequent emergence of prosocial behaviors linked to concern for others (Decety, 2020), offering benefits for social life generally.

As a result, by questioning the expression of empathy in sporting games, we focused on those with several players, each of whom represents a place of intersubjectivity in which the players test themselves through physical contests.

According to many studies, some of which have been presented in this study, bodily games, particularly traditional sporting activities, tend to encourage and strengthen participants’ empathic behavior. If empathetic dispositions are present at an early age (Decety and Holvoet, 2021), exercise through body games would reveal and preserve their full potential. Thus, empathy may be considered a pedagogical construct that can be better handled if the internal and external logics of some classic sports activities are thoroughly investigated. To date, few authors have researched in the field of gaming, in their functioning and in their unique traits (Parlebas, 1995). Are sports, however, the only games that may reveal and encourage empathetic conduct, according to the scientific literature? How can TSG foster educational and social innovation, as well as empathy?

## 4. Empathy improvement through motor interaction games

Many games with high bodily embarrassment, namely, roleplaying or theater games, are inspired by TSGs or resemble them in terms of the relationships between participants. While words can accomplish things (Dugas et al., 2022; Dugas, 2023), “more inclusive bodily engagement is the glue that holds empathetic flow up for an education in empathy to “educate emotions” (Zanna and Jarry, 2019). Thus, games designed for this purpose, particularly TSG, may effectively offer a multitude of educational, social, and societal values, especially when designed by work in the field of motor praxeology (Parlebas, 1981, 1999, 2020). Accordingly, they allow for the discovery of: (1) the development of “creative skills” within communal sporting practices (Obœuf et al., 2020); (2) the reciprocal impact of traditional games and collective sports from the perspective of learning transfer (Parlebas, 2005a; Dugas, 2011b); (3) aggressive or cooperative behavior through the Sitting Ball Game (Obœuf et al., 2008; Dugas, 2011a); and (4) emotions and wellbeing (Lavega-Burgués et al., 2023). Recently, our reflexive path led us to believe that TSG, studied in its intrinsic reality and for itself in an inseparable relationship with the practitioners working within it, opens the door frequently to unnoticed or unknown potentialities, which we have discussed through the examples of games in the following sections.

### 4.1. The bear, the guardian, and the hunters

**Figure 2:**
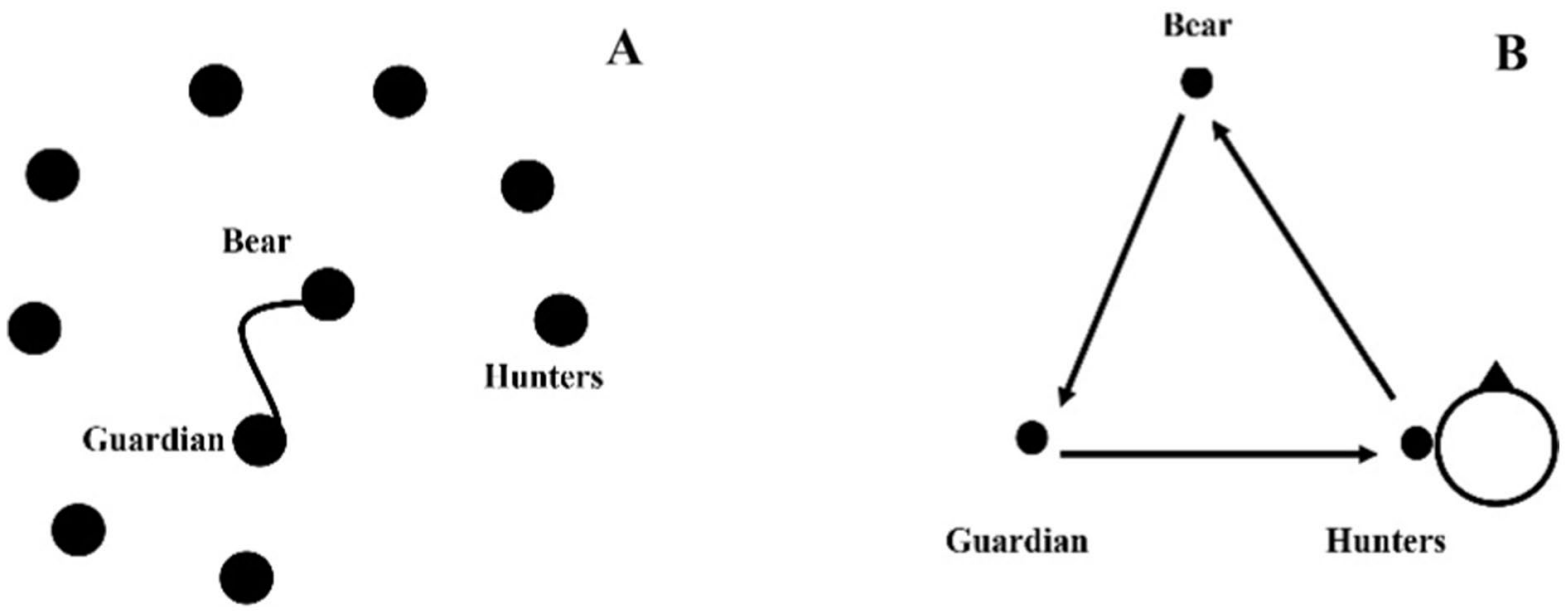
**(A)** Graphic representation of the game “The Bear and its Guardian.” **(B)** Mapping of the network of permutable and interconnected roles in the game using graph theory (Loyer et al., 2015).

The bear, the guardian, and the hunters are examples of three chained roles (Parlebas, 2000; Loyer et al., 2015; Martínez-Santos et al., 2020). According to the game’s internal logic, the guardian’s purpose is to safeguard the bear (who is immobilized with a rope connecting him to his protector) from assaults (hunters). These hunters try to touch the bear as the guard tries to touch the hunters (Figure 2). The players switch roles with each touch: “It is a ternary relationship that mobilizes three individuals with various respective duties and is completed from the guardian to the bear through a hunter” (Parlebas, 2000). Throughout the game, any participant might adopt the three roles. It indicates that a partner may become an opponent (the defender can become a hunter), which means that each player can put himself in the role of the other and experience what he/she experienced. Changes in roles are central to this game. Thus, the interaction network is always changing, and there are no systematic permutations because role changes are related to the players’ immediate successes and failures, their impulsive decisions, and their interpersonal preferences. This style of game is devoid of accountability (no score) and a stopping rule.

Such role changes in sport are certainly implausible since the interactional network is fundamentally stable (a player never changes teams during the game).

For decades, the permuting role changes permitted in TSG have been the structural foundation of non-institutional games focused on empathy, in which bodily engagement is essential, as described in the following.

Observation of players in the games of the Bear, the Guardian, and the Hunters showed that, in this TSG coexperienced body/body resonance game, participants shared emotions (affective arousal and emotional stimulation). Additionally, it demonstrates an empathetic concern (Decety and Cowell, 2014) between the guardian and the bear (motivation to care about the other) by assisting in observing the other in the game and taking behavioral indicators. The following sections may serve as an education in acting through a kind of “interactive building through experience” in this game (Debarbieux, 2008). Overall, the purpose is to reduce violence to improve interpersonal wellbeing and empathy (Favre, 2007).

### 4.2. The four musketeers game

**Figure 3:**
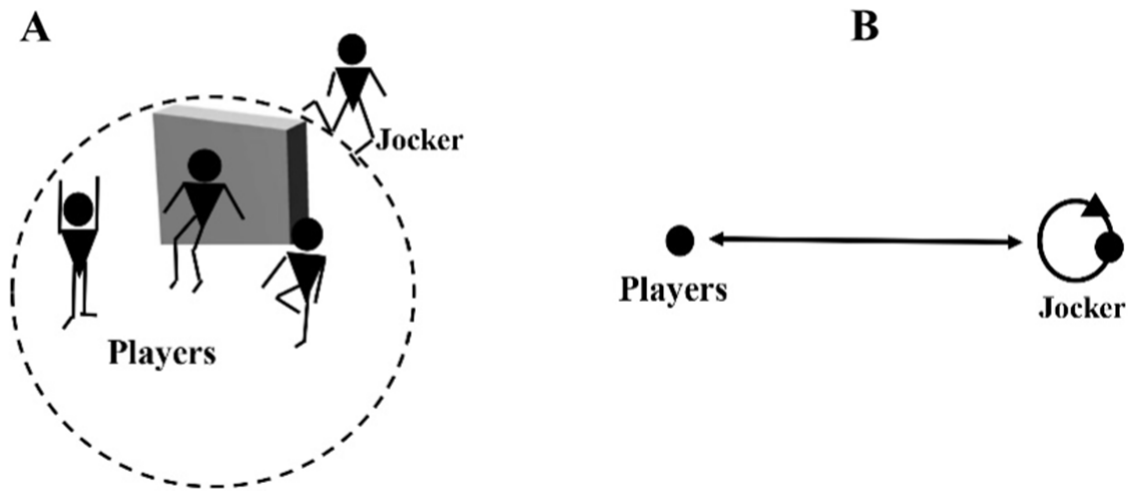
**(A)** Illustration of schematic and **(B)** mapping of the network graph of permutable roles in “The Four Musketeers Game”.

An interactional sports activity known as “the game of the musketeers” was developed by a researcher (Omar Zanna) and a trainer (Bertrand Jarry) and was initially attempted on prisoners to recover the perceived “lost” empathy (Zanna, 2010). The musketeer’s game has also been employed, particularly, with young primary school pupils (7–9 years old). The suggested scenario’s internal logic dictates that the players compete in four-player teams. The first player has his arms spread parallel to the ground, the second to the sky, the third stands on one leg, and the fourth called the joker keeps running around the room on a predetermined path (Figure 3).

If one of the first three players becomes exhausted, the first three players have the right to request that the joker be replaced. As a result, the group that holds its position the longest wins the round. Also, the roles in this game are changing (fluctuating). Thus, players are encouraged to express themselves verbally and physically after each round. The authors state that using these activities in classrooms will promote a positive educational atmosphere. Accordingly, Zanna (2015b) has stated that the idea of a link between facial expression, physical expression, and emotion implies, on the one hand, that emotion translates bodily manifestations, namely, facial (Izard, 1977), and, on the other hand, that these same expressions are indices of the emotion (Tcherkassof, 2008) that one wishes to share with others (Maury, 1993).

The game experience was followed by collective reflexivity, in which students verbalized their feelings and/or acquired knowledge (concerning the empathic concern). The repetition of the game and the verbalization after and between the sessions are seen as reinforcers of the previous experience (increasing motivation to empathize with the other) that allows, over time, to stimulate and comfort empathic perspective sharing.

Indeed, three levers have been extracted from the implementation of the Four Musketeers’ game in schools and colleges and inspired by Decety and Cowell’s (2014) empathy processes:

- The first lever is concerned with emotional awakening and stimulation.
- The second lever is the empathetic concern (motivation to care about one’s partner): To monitor the opponent player in the game and to receive behavioral signals from other musketeers as a team.
- The third lever is perspective-taking through cognitive empathy and social reasoning. It is a question of learning basic life rules as a person and as a citizen in society.

All interactional dynamics in physical engagement betray a fantasy existence by taking on roles, changing roles, and confronting counter-roles.

### 4.3. The school catch–wrestling experiment

Catch–wrestling is a “complicated opposition,” which means that the reproduction of gestures by behaving and acting “as if.” This involves a form of “economic” rivalry in which opponents collaborate depending on the circumstances and opportunities. The player’s behavior is fluctuant, thus, “Sometimes [the player] exceeds the ring’s formal limit and continues to attack an opponent who is legally protected by the ring ropes. Other times, he re-establishes this boundary and demands protection for what he did not respect in the previous minute” (Barthes, 1957). Hence, catch–wrestling requires combat strategies in a complicated relationship between two or more players. It’s a “fighting spectacle” in which one person appears to exert physical dominance over another while the other refuses to express his or her emotions publicly. The catch exercise was given to students with the instructions to break the rules in order to teach them and prepare them for role-playing scenarios.

We presented catch-wrestling in a novel way as a substitute for school wrestling to make it instructive and to build empathy via “teamwork” through physical engagement. Two studies were carried out, the first at a professional high school (Loyer and Dugas, 2014) and the second at a primary school in a priority education zone (Dugas and Loyer, 2018). In both cases, the teacher assisted students in reflecting on potentially “deviant” behaviors to better understand and, maybe, control them. The scenarios presented include an aggressor, a victim, and an observer. Consequently, it was meant to offer a range of collaborative games structured around a subject and to integrate simple motor addressing strategies in groups of four over the course of an eight-session learning cycle (mixed group).

School catch–wrestling was interpreted by us as a traditional (non-institutional) sporting game in which the players establish and manage the rules based on their preferences and feelings. This novel-designed TSG, on the other hand, falls within the category of didactic sporting events, which encompass “all motor situations codified by instructors under educational principles and for instructional aims” (Dugas, 2004). The fundamental purpose of the proposed cach-wrestling game’s internal logic is to keep game morality by proposing a situation in which a “perfect bastard” fights against a “hero,” as recommended by previous research (Barthes, 1957; Loyer and Dugas, 2014). Besides this, the permutable interactional network structure is intended to increase collaboration rather than to combat where gestures “simulate violence.”

According to the findings, second-year students were more competent in expressing themselves verbally and in writing during class. They immediately imagined a fact-based scenario at a disco. The topics of discussion included civic engagement and education, respect for others, gender equality, teamwork, injustice, and friendship, among other things.

Similarly, the instructor suggested some places, things, and scenarios related to intimidation to the youngest primary students (9–11 years old). These young people created an amusing roleplaying game out of short films (animated designs) to combat partner intimidation (Dugas and Loyer, 2018). Thus, the originality of the catch–wrestling game is to simulate violence in a rigorously cooperative game where everything is openly discussed.

The emotional and cognitive components of empathy have been identified and integrated (empathy has matured). Students who witnessed aggressive conduct were given contradictory responses: support the aggressor, be indifferent, and help the victim (in various ways).

To summarize, a few criteria must be respected in school. Catch–wrestling as a path for empathy teaching through corporeal involvement to avoid any emotional misunderstanding that is conducive to hate or opposition to the objectives pursued:

- First, empathic flow through interactions is an ingredient of individual and collective wellbeing when deployed in a controlled, collaborative, supported, and monitored context over time (Curchod-Ruedî et al., 2011).
- Second, in terms of learning input, four phases must be followed, based on specific principles (Zanna and Jarry, 2019): practice together by engaging physically; observe others; switch roles; and talk about feelings by expressing them.

The original school catch–wrestling game, among non-zero-sum games, demonstrates the benefits of moving away from strictly competitive games in terms of empathetic concerns. As a result, the internal logic of the game might potentially fit into an empathy education curriculum.

## 5. Empathy improvement through complex, paradoxical, and digital games

### 5.1. Empathy in the four-corner game

Social interactions in sporting events are both verbal and non-verbal (Parlebas, 1999). As a result, “one participant’s motor activity influences one or more other participants’ behavior visibly” (Parlebas, 1999). This effect may be seen as a function that facilitates the performance of the motor task, where collaboration is encoded as “communication,” while opposition and resistance are processed as “counter-communication.” Considering this, some ludomotor game events, known as paradoxical situations, allow for complex, ambiguous, and ambivalent partnerships (Parlebas, 1999, 2005a,b). As the participants’ contradictory behavior leads to a dialectical background with a rich social, psychological, and educational context, these activities continue to defy classification. The stages of alliance reversal that are the cause of uncertainty may also be identified, and they are viewed as a free strategic choice between cooperation and competition, or even possible apathy toward other participants (Parlebas, 1999). Individual strategy, motor control, and affectivity are all emphasized. Increases in autonomy and decision making are gains in learning in this situation. The investigation of the paradoxical four-corner game, which we present here as an example, was extremely fascinating in this regard.

**Figure 4:**
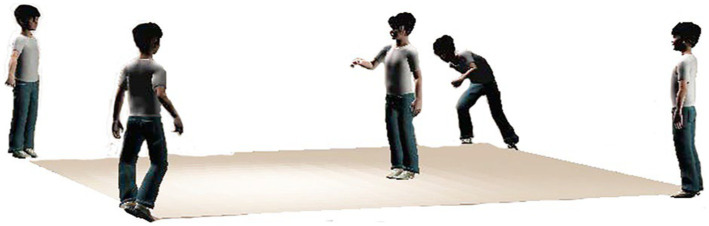
Illustration of the spatial organization of the players in the four-corner game (Ben Ali, 2018).

The four-corner game is well renowned for being played in a 5-m^2^ area (Figure 4). The game usually accommodates five players, one in the middle (central role) and the others in each of the four corners (corner role). The players in the corners attempt to exchange corners at their leisure to avoid being overtaken by the player in the center. The player who is left without a corner occupies the center. Since each player can choose whether to collaborate or to oppose the participants in the other corners, the rules allow for the development of ambiguous or of paradoxical interactions. When two players agree to switch corners in this game, they collaborate by coordinating their efforts.

The internal logic of the game state that, when players dispute on the same corner of choice, corner players are both friends and enemies. Nonetheless, a preliminary analysis may reveal the core connections between rivals (R = rivalry) and partners (S = solidarity).

The four-coin game allows players to become partners or opponents. When one player decides to exchange his corner with another one, the other player decides quickly to move backwards and reclaim his position. If the supposed partner loses his corner during this maneuver, the player who was previously a potential partner becomes a declared rival. The position of one of the players, who was formerly a partner, abruptly transforms from a potential partner to a declared rival when that player becomes restricted. Parlebas (2005b) claimed that analyzing the four-corner game graph (equation 1) results in the formation of a non-exclusive bigraph.


(1) R∩S≠Ø

Any non-verbal cues that corner players share during natural conversation are referred to as non-verbal communication. In the present game involving a complex motor interaction, messages are discreetly sent, and non-verbal cues have highly specific importance (Vinciarelli et al., 2009). This is what upset interest in comparing how playing a paradoxical game affected the empathetic process in young people starting school (primary school) at the age of 6–7 years with young people starting university at the age of 19–20 years (Ben Ali et al., 2020b). The extent of “spatialization” generated by all interactions and the empathy process is at the core of the topic under review. A sociometric analysis of interindividual interactions during the paradoxical four-corner game was produced as a consequence of this research. Two semi-structured interviews were added to the observation, which had already benefited from a quantitative and qualitative analysis of the in-game behaviors and behaviors that could be observed *in situ*, to shed further light on the players’ attitudes and intents. The interviews were carried out once at the end of the game (without watching the scenes) and once more after 15 days with scenes from the players’ game in front of them.

There was a significant difference related to the behavior of players, and it was correlated to their age. In fact, at the youngest ages, the sociometric matrix was significantly correlated with central-player searching behaviors around oneself. Despite common belief, the interviews revealed that older players exhibit less transparency in their actions. Their goals are concealed by their acts. Even better, they do it with awareness. However, they are better able to explain and decrypt the anti-communication goals throughout the viewing process.

In brief, during the traditional four-corner game, young people aged 6–7 years showed more expression of the inter-subject empathy process. Their intentions to give way to the player in the middle were identified as an act of empathy to aid him in playing with them, even though this remained a contradictory behavior in and of itself.

### 5.2. Empathy in sitting ball game

The Sitting Ball Game (SBG) replicates the ambivalence and instability of social life by enabling players to choose their partners and opponents and to change them throughout the game. As a result, this playful organization differs significantly from sporting structures in which ambiguity and instability have no function. According to Obœuf et al. (2008), it is structural uniqueness that will allow individuals to participate in the SBG, regardless of whether their bodies maintain or not the socioaffective connections created in other contexts.

In the SBG (Guillemard et al., 1984), the players are distributed over the playing field and attempt to elude a highly convoluted ball. The movement of the ball is one of the constraints. Thus, players are not assigned to a team, and when they have the ball, they may choose if they want to draw over an opponent or pass for a partner. However, if the player wants to pass the ball to another, he/she has to bounce it on the ground, while a shot on the opponent should be in the air. If the ball touches one of the players, he/she becomes a prisoner and must sit on the ground. He/She must then wait to be released by a participant’s pass (partner) or by changing bounces (from an opponent).

**Figure 5:**
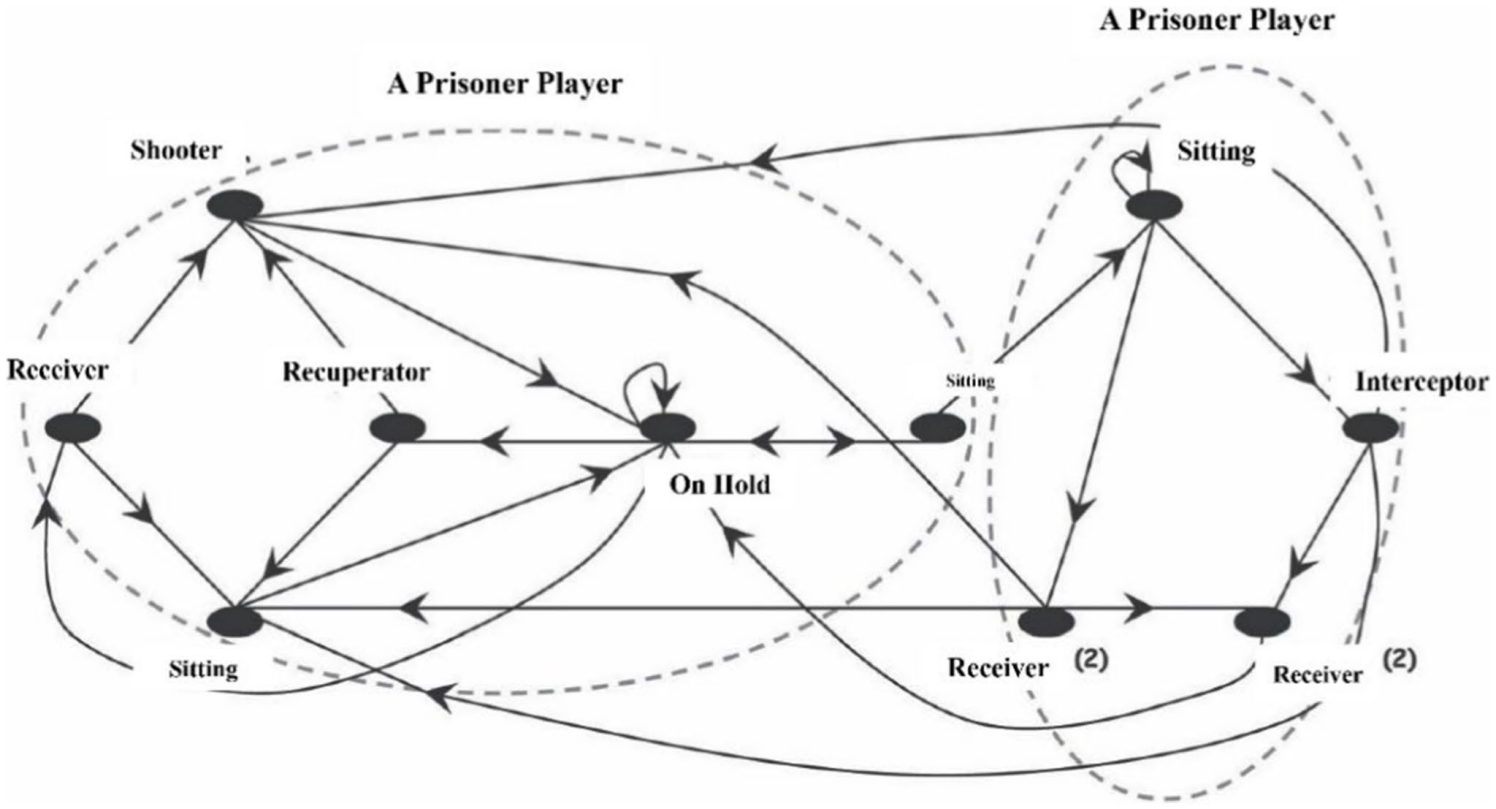
Interaction Graph representation of the Sitting Ball Game (Obœuf et al., 2010).

The rules of the SBG allow for ambiguity and paradox during the game (Figure 5). As a result, the player chooses his or her partners and opponents at any time throughout the game. Thus, he/she has the opportunity to cooperate or oppose at any time. Indeed, one is constantly on the lookout for other players, which is one of the characteristics and levers of empathic availability and emotional education (Zanna and Jarry, 2019). The four steps include physical play, observation, role changes, and verbalization after the fact. These are the constituents that will promote empathy.

The ambivalent interactions (Obœuf et al., 2008; Lavega et al., 2018) lead player interactions into ambiguous or paradoxical situations where each player is potentially both an ally and an opponent of the other players at the same time (Parlebas, 2016). Against this backdrop of contradictory relationships, it is hard to predict the players’ behavior because each one will act following his or her subjective socioaffective preferences at different times during the game (Obœuf et al., 2008). Some researchers argue that we can use surveys to determine the structure of socioaffective relationships as well as the connection of interlink communications by observing them throughout the game (Parlebas, 1999, 2005b; Obœuf et al., 2008). With the use of graph theory, the two networks were then compared (Bornholdt and Schuster, 2001). If the comparison revealed a strong link between these two networks, it implied that the actors made passes to the players they liked the most and the passes were not made to the players they liked the least.

Moreover, “deviants” who take the risk of shooting an in-group member or passing the ball to a member of another group are immediately punished. Then, members of the in-group no longer transfer the ball to them or, worse, they “kill” them to give them time to reflect on their transgression of group norms. Obœuf et al. (2010) noted that “it is the group’s social control over its members that makes these acts (shooting for the in-group or heading for the out-group) look abnormal. As a result, collective authority cannot be challenged.” Indeed, whatever the game, we generally put ourselves “in the place of the other” to understand their viewpoint on the forthcoming action and anticipate their reaction. Obœuf et al. (2010) stipulated that “This empathy is highly dependent on inferential mechanisms (Bromberg, 2004), i.e., the context (Bateson, 1951), or internal logic (Parlebas, 1981, 1991, 1999).” In the case of the “sitting ball,” where empathetic acuity plays a major role, it allows escaping. Some games do have an unanticipated socioaffective coloration (Obœuf et al., 2008). In terms of socialization, the distinction between the SBG and institutional sports is significant. The forms of communication and meta-communication generated by the game’s internal logic provide insights into the subtle pathways via which socializing occurs.

In other words, some individuals are more skilled than others at predicting the intentions of others; in a nutshell, they have greater empathy. As stated by Obœuf et al. (2010), “there is a superposition of protagonists in ‘SBG’ who best perceive the choices of others at the socioaffective level (sociometric questionnaire) and those who show themselves to be the most effective at the instrumental level, having the most developed dodging abilities.”

### 5.3. Empathy in video, “Phygital,” or digital games

We are concerned about the future of traditional sporting games, but we cannot dismiss the technological advancements that are infiltrating the daily lives of humans, particularly children. As a result, in the face of a dazzling expansion of digital and “phygital” games, bodily involvement and physical interaction are frequently challenged. While physical games promote social and emotional interaction by requiring the physical presence and engagement in a “face-to-face” situation (Parlebas and Schmitt, 1975; Tisseron, 2011; Zanna, 2015b; Dugas and Loyer, 2018; Zanna and Jarry, 2019), they also assist with the joy of acting, commitment, and, for some, the development of empathic skills, particularly on the affective-emotional side. However, what about more distant games such as digital/video games and virtual reality (VR) games?

Meta-analyses demonstrate that, depending on the degree of technology immersion, the development of empathy is varied, but always superior to basic frequently communicated information (Herrera et al., 2018). Indeed, the conventional definition of empathy is to put oneself in the position of another. If this perspective enhances participants’ empathy, the effect is smaller as compared to technology, especially if the virtual/digital world is immersive. Finally, several meta-analyses devoted to empathy (Tassinari et al., 2021) have shown that, across all kinds of media used, and under particular situations, VR with the embodiment of a virtual character produces better effects (Ventura et al., 2020). This idea has been supported by examples of intergroup interactions. Paradoxically, computer-mediated communication can have an impact on the participants’ empathetic capacity (Nicovich et al., 2005).

To summarize, regardless of the media used (textual, virtual, digital, or others) to improve empathy that helps us live more harmoniously together, the closer we come to physical reality, the more substantial its impact appears to be. The more we engage the participants’ senses (visual, auditory, and proprioceptive systems) and copresence, the more realistic and sustained the impact seems. The current state of the art permits us to hypothesize that, in the context of “phygital” games (escape/serious games). The more significantly individual’s body is sollicited, the higher the degree of his motor involvement. Furthermore, the more immersive the media, the closer we are to a realistic scenario, and the greater the influence on empathy, assuming that the empathetic mechanism is effectively mastered. For example, researchers simulate blindness by covering the participants’ eyes. Despite evoking demonstrable “empathic care,” the situation has resulted in misinformation and reinforced stereotypes (Silverman, 2015). It is not a question of bringing into play persons who experience otherness to generate, stimulate, or induce empathy. Empathic care necessitates a pedagogical effort, which can be enhanced by the use of conventional or comparable sporting games.

## 6. Conclusion

In this study, we attempted to emphasize the educational significance of traditional sporting games. The major goal of the study was to demonstrate how these games provide a diverse spectrum of human interactions that enhance availability and empathic attitudes.

Traditional sporting games have numerous varieties that allow players to be both actors and authors of their interactions. Indeed, the ludomotor face-to-face interactions create care for others, and when one pays attention to the other (empathic awareness), the intention to help the other (empathic motivation) is stronger. This is the cornerstone of relational empathy education.

As a result, we attempted to broaden the domains of the praxeological and structural analysis of TSGs by employing the prism of original empathy in Section 3 of this article. Furthermore, examining the interaction systems through the prism of the internal logic of the games helps us better comprehend the “I,” “you,” and “we” that combine to form emotional connections. According to Parlebas (1981), there was “I” in the “game,” but there was also a “game” in the “I.”

Moreover, many research studies, some of which have been discussed in this article, reported that body games, particularly traditional sports activities, typically boost and reinforce players’ empathic behavior. Thus, if empathic dispositions are present at a young age (Decety and Holvoet, 2021), exercise through body games would uncover and preserve their full potential. Such is the case with paradoxical games, which offer further decision-making flexibility than institutionalized games. This analogy is explained by Pierre Parlebas, who argues that, in sports, “what counts is what counts.” From this point of view, there is less prospect of socioaffective interactions interfering significantly with the sports gaming process. They undoubtedly play a role, but it is measured. The internal logic is a manifold of efficiency, and friendship gives way to operationality (Obœuf et al., 2010).

As a result, it is easier to conceive a mutual help connection in TSGs (Reynaud and Richebé, 2009). These motor situations imply that players dare to interact together by participating with themselves since when you join a game, you are doing it for the pleasure of moving, interacting, and doing things together. Therefore, we believe that these games provide educators with several opportunities to contribute to education in empathy and its associated dimensions through their internal logic coupled with didactic processing. The concept of the school catch-wrestling game, presented in Section 3 of this article, would be an outstanding demonstration of a scenario capable of bringing into action collective dedication amid difficult circumstances. In this sense, Parlebas (1975) specifies that “despite what tenacious tradition maintains, gambling is an activity that is neither free, nor disinterested, nor sterile.” Nonetheless, this playful face-to-face interaction remains a second-degree “genuine fiction” in which the player immerses himself in the same seriousness as in reality (Brougère, 2005). Additionally, first-person observation of the game deviates from its original meaning, generating a confessional circle around phrases such as labor, learning, or education. In short, games give amusement while also having the ability to be instructional, favorable to learning, and even teach empathy.

In terms of perspectives, traditional sporting games can bring a variety of educational, social, and societal benefits, especially concerning work relating to motor praxeology (Parlebas, 1981, 1999, 2020). Indeed, they allow researchers to learn about (1) the development of “creative skills” within team sports practices (Obœuf et al., 2020); (2) the reciprocal impact of traditional games and team sports on learning transfer (Parlebas, 2005a; Dugas, 2011b); (3) aggressive or cooperative behavior through the SBG (Obœuf et al., 2008; Dugas, 2011a); and (4) emotions and wellbeing (Lavega-Burgués et al., 2023). Finally, our reflective analysis led us to believe that a TSG, studied in its inherent reality and for itself in an inseparable relationship with the practitioners who work within it, opens the door to potentialities often unnoticed or passed over in silence.

## Data availability statement

The original contributions presented in the study are included in the article/supplementary material, further inquiries can be directed to the corresponding author.

## Author contributions

ED and BB actively and equally participated in the design of the study and in shaping the final version of the manuscript. All authors contributed to the article and approved the submitted version.

## Conflict of interest

The authors declare that the research was conducted in the absence of any commercial or financial relationships that could be construed as a potential conflict of interest.

## Publisher’s note

All claims expressed in this article are solely those of the authors and do not necessarily represent those of their affiliated organizations, or those of the publisher, the editors and the reviewers. Any product that may be evaluated in this article, or claim that may be made by its manufacturer, is not guaranteed or endorsed by the publisher.
